# *Stevia rebaudiana* extract (main components: chlorogenic acid and its analogues) as a new safe feed additive: evaluation of acute toxicity, sub chronic toxicity, genotoxicity, and teratogenicity

**DOI:** 10.3389/fvets.2025.1646665

**Published:** 2025-09-04

**Authors:** Yuting Li, Liping Zhu, Dongsheng He, Ling Fang, Yajing Li, Shusheng Tang

**Affiliations:** ^1^National Key Laboratory of Veterinary Public Health and Safety, College of Veterinary Medicine, China Agricultural University, Beijing, China; ^2^Zhucheng Haotian Pharm Co., Ltd, Zhucheng, China

**Keywords:** *Stevia rebaudiana* extract, oral acute toxicit, sub chronic toxicity, genotoxicity, teratogenicity

## Abstract

**Introduction:**

*Stevia rebaudiana* extract (SREC), primarily composed of chlorogenic acid and its analogues, is a promising feed additive with potential benefits for livestock performance, gut health, and antioxidant capacity. However, its safety evaluation has not been comprehensively studied.

**Methods:**

The safety of SREC was assessed through a series of tests, including acute oral toxicity in mice and rats, a 90-day subchronic toxicity test in rats, genotoxicity assays (Ames test, mouse bone marrow micronucleus test, and mouse sperm abnormality test), and teratogenicity evaluation in pregnant rats.

**Results:**

The acute oral toxicity test indicated that the LD₅₀ of SREC in mice and rats was greater than 5,000 mg/kg body weight. In the 90-day subchronic toxicity test, SREC was non-toxic at doses up to 50,000 mg/kg in feed. The Ames test showed no mutagenic effects on *Salmonella typhimurium strains* TA_97_, TA_98_, TA_100_, and TA_102_. No genotoxicity was observed in the mouse bone marrow micronucleus test or the sperm abnormality test, with no significant differences compared to controls (*p* > 0.05). Similarly, no teratogenic effects were found in pregnant rats, with normal embryonic development across treatment and control groups.

**Discussion:**

SREC exhibited low toxicity in both acute and subchronic tests, and no evidence of genotoxicity or teratogenicity was observed. These findings suggest that SREC is safe as a potential feed additive and provide valuable reference data for its safety evaluation.

## Introduction

In recent years, the rapid development of the livestock (1) and poultry farming industry, alongside advances in feed technology, has led to the widespread use of antibiotics and growth promoters in commercial compound and complete feeds (2) to enhance growth performance and prevent diseases. However, the excessive use of antibiotics resulted in the emergence of antibiotic-resistant bacteria, posing serious threats to both animal health and human health through the food chain, which in turn compromises the effectiveness of clinical antibiotics (3). Consequently, many countries and regions have restricted or banned the use of antibiotic growth promoters in animal husbandry, accelerating the shift toward natural, safe, and effective alternatives (4–6). The demand for plant-derived feed additives with functional properties continues to grow.

*Stevia rebaudiana* (7) is traditionally cultivated for its sweet-tasting compounds. In addition to the well-known steviosides, stevia leaves are also rich in chlorogenic acid (CGA) and its analogues (8), which are phenolic compounds with a wide range of biological activities, including antioxidant (9), anti-inflammatory (10), antimicrobial (11), antitumor (12), antihyperuricemic (13), immunoregulatory, and anti-fatigue (14) effects. Leveraging these properties, we developed a CGA-rich extract from stevia leaves, hereafter referred to as SREC, with a CGA content of approximately 43%. Unlike conventional stevia sweeteners, SREC is specifically formulated to enhance the functional benefits of CGA and its analogues. Given its demonstrated bioactivities, SREC represents a valuable alternative to antibiotic growth promoters in livestock and poultry farming, with the potential to improve animal health.

However, despite its promising bioactivity, toxicological studies on SREC are limited, particularly in the context of its use as a feed additive. Establishing the toxicological safety of SREC is a critical prerequisite for its approval and application in animal nutrition. Without sufficient toxicity data, the use of SREC will face regulatory and safety challenges, hindering its practical implementation. Therefore, the present study aims to conduct a comprehensive toxicological evaluation of SREC, including assessments of acute oral toxicity, sub-chronic toxicity, genotoxicity (Ames test, mouse bone marrow micronucleus test, and mouse sperm abnormality test), and teratogenicity. These findings will provide essential scientific evidence to support the safe and effective integration of SREC into livestock feed and promote its development as a natural alternative to antibiotic growth promoters.

## Materials and methods

SREC (containing 43.3% total chlorogenic acid), was provided by Zhucheng Haotian Pharm Co., Ltd. Sodium azide (NaN_3_, 99.9%) and dicofol (98.1%) were purchased from Sigma Aldrich, USA. 2-Aminofluorene (2-AF, 98%) was obtained from Alfa Aesar, UK. Formaldehyde (37–40%) was purchased from Tianjin Damao Chemical Reagent Factory, China. Potassium hydroxide was sourced from Shantou Xilong Chemical Factory Co., Ltd., China. Eosin and alizarin red were obtained from Beijing Chemical Reagent Company, China. Hematoxylin was purchased from Beijing JiuZhou Bolein Biotechnology Co., Ltd., China. Paraffin was obtained from Leica Biosystems.

The following chemicals were all purchased from Sinopharm Chemical Reagent Co., Ltd., Beijing, China: potassium dihydrogen phosphate, disodium hydrogen phosphate, methanol, glycerol, Giemsa stain, cedarwood oil, ammonium phosphate, citric acid, dipotassium phosphate, magnesium sulfate heptahydrate, sodium chloride, potassium chloride, sodium hydroxide, hydrochloric acid, sodium dihydrogen phosphate, D-biotin, L-histidine, glucose, glucose-6-phosphate, coenzyme II, agar powder, nutrient agar, nutrient broth, glacial acetic acid, glycerin, chloral hydrate, neutral gum, xylene, anhydrous ethanol, and aqueous eosin.

Blood biochemical test reagents, including serum albumin (Alb), alanine aminotransferase (ALT), aspartate aminotransferase (AST), blood urea nitrogen (BUN), total cholesterol (TCH), creatinine (Cr), glucose (Glu), and total protein (TP), were purchased from Shanghai Kehua Bio-engineering Co., Ltd.

### Animals and care

SD rats and ICR mice (both female and male) of SPF grade were purchased from SBF (Beijing) Biotechnology Co., Ltd. The SD rats had body weights ranging from 180 to 220 g, and the ICR mice had body weights ranging from 18 to 22 g. All SD rats and ICR mice were housed for 7 days at the Animal Drug Safety Evaluation Center of the Ministry of Agriculture (Beijing) to acclimate to the standard laboratory conditions of room temperature (20–26 °C), relative humidity (50–65%), and artificial lighting (12-h light/dark cycle). During the entire experimental period, all SD rats and ICR mice were provided with sufficient standard rodent chow and water, and were allowed to eat and drink freely. All facilities were maintained to prevent contamination from exogenous factors, ensuring cleanliness throughout the feeding and experimental process. The experimental protocols involving animals were approved by the Institutional Animal Care and Use Committee of China Agricultural University, adhering to ethical standards for the humane treatment of animals (Animal Ethics Approval No. AW80804202-2-1).

#### Acute oral toxicity study

Our preliminary study indicated that a single oral dose of SREC at levels of 512, 1,600 and 5,000 mg/kg · body weight (bw) did not cause any toxic effects when administered to both rats and mice during the preliminary phase. Based on these results, a dose of 5,000 mg/kg·bw was selected for further testing, following the principles of the OECD Test Guideline 425 (15) (Up-and-Down Procedure), which allows for testing doses up to 5,000 mg/kg·bw to determine the toxicological limit dose (16, 17). For the formal test, 30 rats and 30 mice were used, with the rats and mice each randomly divided into 3 groups of 10 animals each (5 males and 5 females per group). The test substance was prepared as a suspension in 1% carboxymethyl cellulose sodium and administered orally. All animals were observed for general behavior, toxic symptoms, and mortality over a period of 7 days. If any deaths occurred after 4 days of administration, the observation period was extended to 14 days, and if necessary, up to 28 days. Necropsies were performed on any deceased animals, and the results were documented. At the end of the experiment, surviving animals underwent gross necropsy, with observations recorded for mortality rate, clinical symptoms, body weight changes, and general examination results throughout the observation period.

#### Sub chronic toxicity study

Eighty rats, aged 4–5 weeks and weighing 70–90 g, were randomly divided into four groups, each comprising 20 rats (10 males and 10 females). The rats were fed diets containing SREC at dosages of 0 mg/kg feed (negative control: NC), 2,000 mg/kg feed (low dose), 10,000 mg/kg feed (medium dose), and 50,000 mg/kg feed (high dose). The feed, sourced from Xiaoshuyoutai Biotechnology Co., Ltd., was thoroughly mixed with the SREC and pelleted. Over a period of 90 days, the health status of both control and experimental groups was monitored daily, assessing clinical signs of toxicity such as changes in behavior, physical appearance, and mortality (18). Food intake and body weight recorded every 5 days at a consistent time to minimize variability. On day 45 and 90, five male and five female rats from each group were randomly selected for hematological and biochemical analysis. Blood samples were collected via abdominal aorta under anesthesia, followed by immediate euthanasia for organ collection and then followed by weighing and dissection. Hematological parameters, including hemoglobin (HGB), red blood cells (RBC), white blood cells (WBC), platelets (PLT), hematocrit (HCT), eosinophils (EOS), basophils (BAS), neutrophils (NEU), monocytes (MO), and lymphocytes (LYM), were measured using a HEMAVET 950FS hematology analyzer (Drew Scientific). Biochemical parameters such as albumin (Alb), alanine aminotransferase (ALT), aspartate aminotransferase (AST), blood urea nitrogen (BUN), total cholesterol (TCH), creatinine (Cr), glucose (Gl), total protein (TP), and triglycerides (TG) were measured using the ELLIPSE automated biochemistry analyzer (Vital Scientific) with specific reagent kits. Fresh organs were collected for organ coefficient calculation and histopathological examination. Organs, including liver, spleen, lung, heart, kidney, and intestine, were stained with hematoxylin and eosin (HE) for pathological observation (19).

#### Salmonella reverse mutation (Ames) test

Following the method described by Hayashi et al. (20), a bacterial reverse mutation assay was performed to determine the genotoxic potential of SREC. Salmonella typhimurium strains (21) TA_97_, TA_98_, TA_100_, and TA_102_, obtained from the National Key Laboratory of Veterinary Public Health Safety, China Agricultural University, were used. Toxicity and solubility (precipitation) of SREC were assessed on all strains (22). Experimental setup: negative control (NC) and positive controls (PC): 2-aminoanthracene (2-AF, 10.0 μg/100 μL) for strains with S9-mix, dexon (50.0 μg/100 μL) for strains without S9-mix, and sodium azide (NaN_3_, 1.5 μg/100 μL) for strain TA_100_ without S9-mix. Dose groups: 30 μg/plate, 6 μg/plate, 1.2 μg/plate, 0.24 μg/plate, 0.048 μg/plate, negative control (Dimethyl sulfoxide), and positive control. Test Procedure: 0.1 mL of test substance (dissolved in Dimethyl sulfoxide), 0.1 mL of overnight bacterial culture, and 0.5 mL of S9-mix or phosphate buffer were mixed with 2 mL of molten top agar and poured onto minimal glucose agar plates. Plates were incubated at 37 ± 2 °C for 48–72 h, and revertant colonies were counted. Mutagenicity was indicated by a doubling of revertant colonies compared to the control or a dose-dependent increase. Each treatment was done in duplicate.

#### Micronucleus test on bone marrow cells of mice

The *in vivo* genotoxic potential (23) of SREC was evaluated using the bone marrow micronucleus test in ICR mice, based on the method described by Hayashi et al. (24). A total of 80 ICR mice (40 males and 40 females) were divided into five groups of 16 mice each (8 males and 8 females per group). Three dose groups were established: 5,000, 2,500, and 1,250 mg/kg·bw. Preliminary toxicity tests showed that the highest dose of 5,000 mg/kg·bw did not induce any clinical signs of toxicity, aligning with the OECD Test Guideline 474 for genotoxicity testing which allows for such dosing if no severe toxicity is observed (25). The SREC was dissolved in a 1% CMC-Na solution and administered by oral gavage once daily for two consecutive days. The negative control group received 1% CMC-Na solution, while the positive control group received cyclophosphamide (40 mg/kg·bw) via intragastric administration 24 h before sacrifice. Observations were conducted at 0.5, 2.5, 5, and 24 h after the first dose, and at 0.5, 2.5, and 24 h after the second dose to detect any toxic effects and treatment-related discomfort. After the final treatment, the mice were euthanized using CO_2_ gas.

Bone marrow preparations were made according to the method of Schmid (26). Micronuclei were identified as small round or oval bodies, approximately 1/5 to 1/20 the diameter of polychromatic erythrocytes (PCE). For each animal, the number of micronucleated cells in 1,000 PCEs was counted, and the ratio of PCE to normochromatic erythrocytes (NCE) was scored in every 200 erythrocytes to assess the toxic effects of the extract on bone marrow cells and hematopoiesis.

#### Sperm abnormality test in mice

Fifty 6-8-week-old rats were randomly divided into five groups. The experimental groups received SREC at 1,250, 2,500, and 5,000 mg/kg·bw (1 mL/100 g·bw). The positive control group (PC) received cyclophosphamide at 40 mg/kg·bw, and the negative control group (NC) received a 1% CMC-Na solution (1 mL/100 g·bw). All substances were administered by gavage once daily for 5 days, prepared as suspensions in a 1% CMC-Na solution. Sperm abnormality tests are conducted in accordance with standard methods (27). On the 35th day after the first treatment, five mice from each group were randomly selected. After euthanasia, both epididymides were isolated, placed in 2 mL of saline, and dissected. The saline containing sperm was aspirated, agitated, and allowed to stand for 5 min. The mixture was then filtered to remove tissue debris, and two drops of filtrate were placed on a slide for each mouse, creating four slides per mouse. The slides were air-dried, fixed with methanol for 10 min, stained with 2% eosin for 50 min, rinsed with water, and air-dried.

#### Teratogenicity test in rats: reproductive and embryotoxicity test design

A total of 120 rats, aged 7–8 weeks, were used in this study, including 80 females (weighing 220–250 g) and 40 males (weighing 250–300 g). The male-to-female ratio was set at 1:2 per cage. Female rats with sperm present in their vaginal smears were identified as newly “pregnant” rats (minimum of 12) and then these pregnant rats were randomly divided into four groups. SREC was prepared as a suspension in a 1% CMC-Na solution. The experimental groups received SREC at doses of 312.5 mg/kg·bw (low dose), 1,250 mg/kg·bw (medium dose), and 5,000 mg/kg·bw (high dose) by gavage (1 mL/100 g·bw). The negative control group (PC) received 1% CMC-Na solution (1 mL/100 g·bw). All pregnant rats were treated continuously for 10 days, from the 7th to the 16th day of gestation. On the 20th day of gestation, the rats were dissected to assess reproductive function indicators, embryonic development indicators, and fetal malformation indicators (28, 29).

#### Statistical analysis

Data were analyzed using GraphPad Prism version 9.0 (GraphPad Software, San Diego, CA, USA). All values are expressed as the mean ± standard deviation (SD). Statistical comparisons between groups were performed, with a *p*-value of < 0.05 considered statistically significant.

## Results

### Acute oral toxicity

During the 14-day observation period, no clinical symptoms or toxic deaths were observed in either male or female rats and mice treated with SREC. At the scheduled necropsy, no abnormal pathological findings were detected in the lungs, spleen, heart, liver, kidneys, or gastrointestinal tract of rats (both sexes) administered 5,000 mg/kg body weight of SREC.

### Sub chronic toxicity study

**Figure 1:**
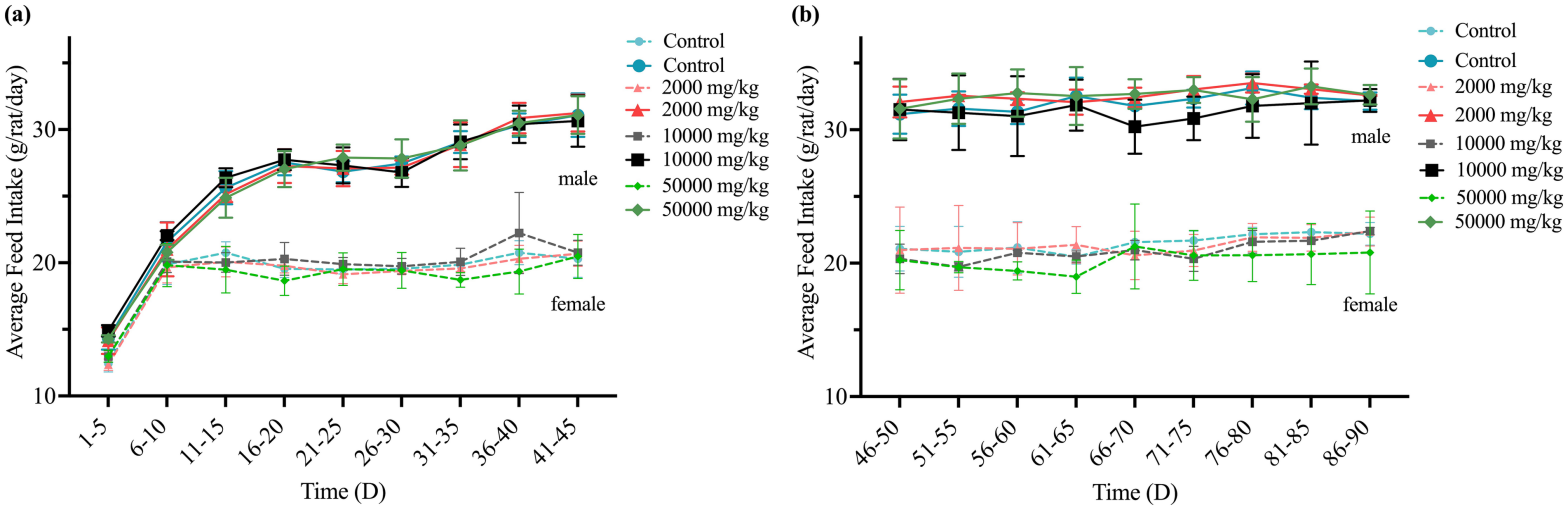
Effect of SREC on average feed intake of rats in the 90-day feeding study, as follows: **(a)** 1–45 days feed intake, **(b)** 46–90 days feed intake.

**Figure 2:**
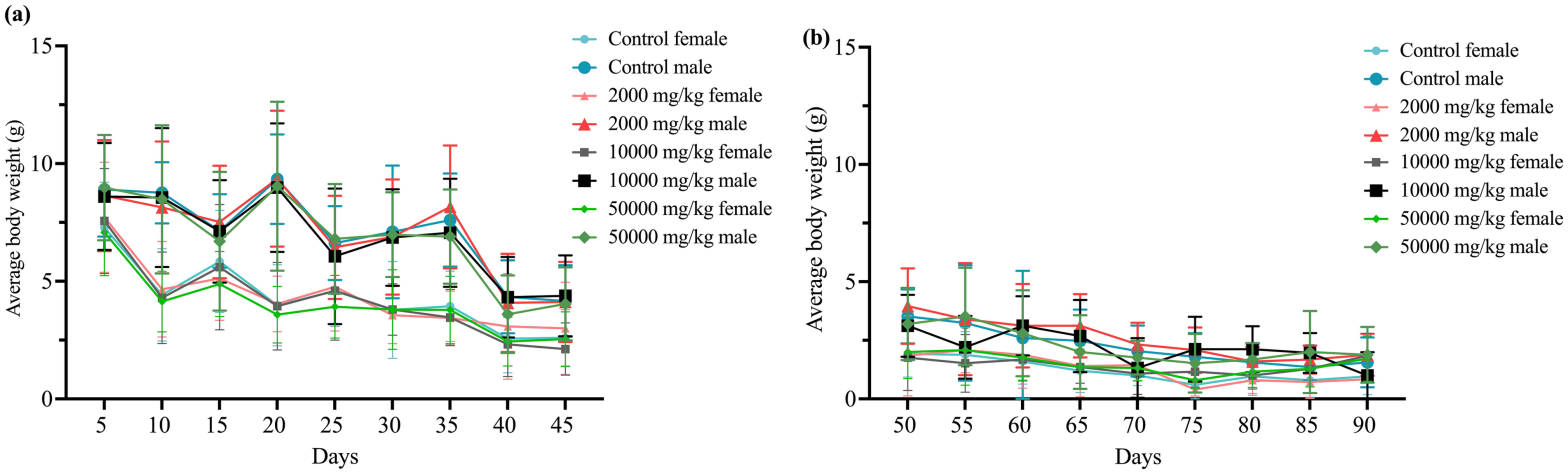
Effect of SREC on average body weight of rats in the 90-day feeding study, as follows: **(a)** 1–45 days body weight, and **(b)** 50–90 days body weight.

Throughout the 90-day study, rats in all dose groups (2,000–50,000 mg/kg feed) and the negative control group exhibited no abnormal clinical signs, including changes in behavior, appearance, excreta, or skin. Feed intake, water consumption, and body weight gain remained stable, with no significant differences among groups (Figures 1, 2 and Supplementary Figure S1).

Hematological evaluations on Days 45 and 90 revealed no dose-related changes in parameters such as hemoglobin (HGB), red blood cell count (RBC), and white blood cell count (WBC); all values remained within normal physiological ranges (Table 1 and Supplementary Table S1). Similarly, biochemical parameters—including albumin (Alb), AST, total cholesterol (TCH), creatinine (Cr), glucose (Glu), triglycerides (TG), total protein (TP), and blood urea nitrogen (BUN)—were unaffected (Table 2). A statistically significant increase in ALT levels (*p* < 0.05) was observed in high-dose females; however, values remained within normal limits, suggesting no clinical relevance.

**Table 1:** Feeding SREC for 90 days: effects on hematological parameters in SD rats.

Groups (mg/kg) feed	HGB(g/L)		RBC (10^12^/L)		WBC (10^9^/L)		PLT (10^9^/L)		HCT (%)		EOS (10^9^/L)		BAS (10^9^/L)		NEU(10^9^/L)		MO (10^9^/L)		LYM(10^9^/L)	
	**♀**	**♂**	**♀**	**♂**	**♀**	**♂**	**♀**	**♂**	**♀**	**♂**	**♀**	**♂**	**♀**	**♂**	**♀**	**♂**	**♀**	**♂**	**♀**	**♂**
50,000	166.80 ± 4.55^*^	168.80 ± 12.36	7.49 ± 0.18	8.30 ± 0.61	6.85 ± 1.49	12.10 ± 2.60^*^	874.80 ± 71.63	763.401 ± 84.37	50.04 ± 1.71^*^	50.24 ± 3.96	0.06 ± 0.02	0.05 ± 0.04	0 ± 0	0.01 ± 0.01	1.79 ± 0.37^*^	4.24 ± 0.92	0.51 ± 0.12^*^	1.15 ± 0.29^*^	4.50 ± 1.03	6.64 ± 1.95
10,000	142.20 ± 13.14	151.20 ± 18.82	6.97 ± 0.54	7.85 ± 0.71	7.40 ± 3.36	10.28 ± 0.74^*^	732.40 ± 72.68	694.60 ± 107.90	45.22 ± 3.50	45.98 ± 4.53	0.04 ± 0.02	0.02 ± 0.02	0 ± 0	0 ± 0	2.76 ± 1.60	3.37 ± 0.33	0.62 ± 0.43	0.68 ± 0.23	3.98 ± 1.46	6.19 ± 0.37
2000	150.80 ± 10.66	165.60 ± 6.77	7.87 ± 0.40	8.72 ± 0.53	5.66 ± 1.77	7.32 ± 1.12	740.20 ± 48.15	619.80 ± 103.39	46.80 ± 3.22	47.81 ± 2.07	0.03 ± 0.02	0.02 ± 0.02	0 ± 0	0 ± 0	1.93 ± 0.68	2.52 ± 0.42	0.37 ± 0.15	0.55 ± 0.10	3.32 ± 1.09	4.23 ± 1.05
NC	155.20 ± 7.16	153.60 ± 9.40	7.80 ± 0.59	8.43 ± 0.68	5.96 ± 0.71	7.01 ± 1.93	784.00 ± 81.41	791.20 ± 77.48	45.48 ± 1.96	45.08 ± 3.04	0.06 ± 0.03	0.06 ± 0.06	0 ± 0	0.01 ± 0.01	2.48 ± 0.41	3.54 ± 1.38	0.32 ± 0.08	0.42 ± 0.18	3.11 ± 0.46	2.99 ± 0.68

*Significantly different from the NC group (*p* < 0.05). **♀-**female**, ♂-**male.

**Table 2:** Effects of SREC on blood biochemical parameters in SD rats after 90 days of feeding.

Groups (mg/kg) feed	Alb (g/L)		ALT (U/L)		AST (U/L)		TCH (mmol/L)		Cr (μmoL/L)		Glu (mmol/L)		TG (mmol/L)		TP (g/L)		BUN (mmol/L)	
	♀	♂	♀	♂	♀	♂	♀	♂	♀	♂	♀	♂	♀	♂	♀	♂	♀	♂
5,000	42.80 ± 1.46	40.38 ± 1.81	44.40 ± 8.17^*^	54.20 ± 10.94	99.40 ± 13.11	102.20 ± 11.67	2.33 ± 0.19	2.19 ± 0.33	32.26 ± 4.37	32.04 ± 7.33	8.00 ± 1.08	8.43 ± 2.48	0.62 ± 0.11	1.26 ± 0.49	67.84 ± 2.94	66.78 ± 4.43	5.75 ± 1.22	5.79 ± 0.57
1,250	42.60 ± 3.05	39.50 ± 1.22^*^	41.20 ± 1.92	52.40 ± 6.43	94.80 ± 11.14	118.20 ± 21.46	2.12 ± 0.21	1.91 ± 0.20	29.08 ± 13.32	34.25 ± 4.37	8.49 ± 2.82	7.76 ± 2.57	0.68 ± 0.36	0.78 ± 0.28	68.32 ± 5.37	65.14 ± 2.45	6.47 ± 1.18	5.26 ± 0.39
312.5	44.04 ± 2.14	40.50 ± 1.36	49.40 ± 10.36	54.80 ± 7.79	102.60 ± 24.56	121.00 ± 37.66	2.11 ± 0.41	1.88 ± 0.47	36.35 ± 1.47	30.52 ± 13.82	7.70 ± 1.10	9.81 ± 2.59	0.56 ± 0.23	0.55 ± 0.20	70.58 ± 5.49	66.96 ± 3.41	6.02 ± 5.91	6.30 ± 0.74
NC	44.76 ± 2.45	41.38 ± 0.68	46.40 ± 3.91	60.00 ± 5.96	108.00 ± 13.17	108.20 ± 15.02	2.24 ± 0.21	1.66 ± 0.53	40.08 ± 3.93	33.84 ± 5.07	7.32 ± 1.47	7.82 ± 1.06	0.59 ± 0.11	0.72 ± 0.38	72.50 ± 4.61	66.40 ± 2.06	6.00 ± 1.10	5.71 ± 1.10

*Significantly different from the NC group (*p* < 0.05). **♀-**female**, ♂-**male.

**Figure 3:**
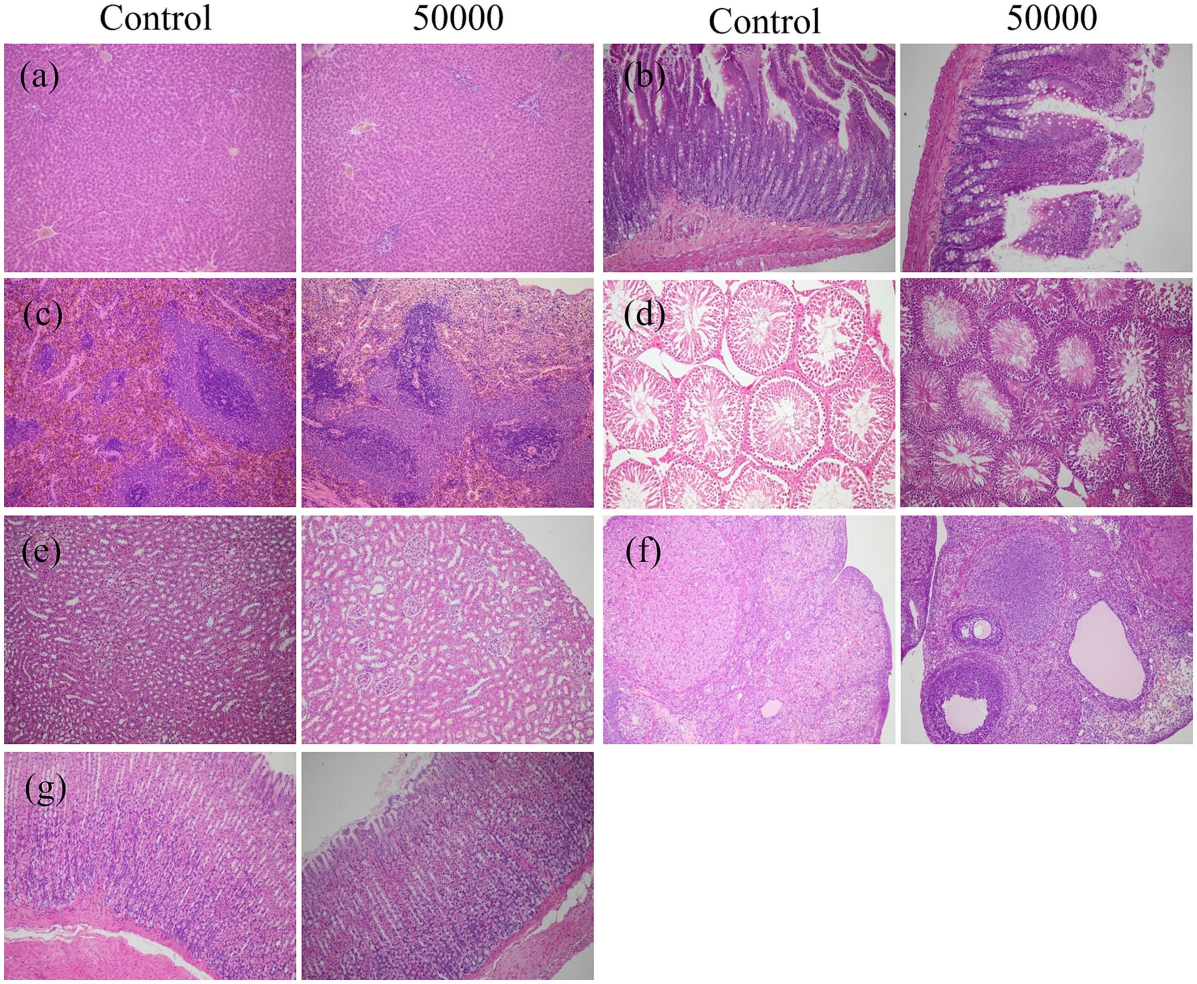
Effect of SREC on the histopathological change in rats after feeding for 90 days (female and male were similar, only the control group and the high-dose group were included); **(a)** liver; **(b)** intestine; **(c)** spleen; **(d)** testis; **(e)** kidney; **(f)** ovary; **(g)** stomach (All images taken at ×20 magnification).

Organ coefficient measurements and histopathological analyses of major organs (heart, liver, kidneys, spleen, lungs, gastrointestinal tract, testes, and ovaries) showed no treatment-related abnormalities at either time point (Table 3, Figure 3, Supplementary Table S3, and Supplementary Figure S2). No signs of hemorrhage, edema, inflammation, or tissue damage were detected.

**Table 3:** Effect of SREC on organ coefficient of rats after feeding for 90 days.

Groups (mg/kg feed)	Liver		Kidney		Spleen		Gastrointestinal		Lung		Heart		Testicle	Ovary
	♀	♂	♀	♂	♀	♂	♀	♂	♀	♂	♀	♂		
5,000	3.61 ± 0.33	3.40 ± 0.18	0.59 ± 0.02	0.62 ± 0.04	0.22 ± 0.04	0.20 ± 0.02	9.99 ± 0.54	7.66 ± 1.14	0.69 ± 0.06	0.57 ± 0.24	0.32 ± 0.06	0.29 ± 0.03	0.74 ± 0.05	0.04 ± 0.01
1,250	3.82 ± 0.78	3.43 ± 0.47	0.64 ± 0.06	0.64 ± 0.08	0.23 ± 0.04	0.20 ± 0.02	9.60 ± 1.91	7.35 ± 0.10	0.73 ± 0.18	0.51 ± 0.05	0.34 ± 0.05	0.32 ± 0.02	0.73 ± 0.06	0.04 ± 0.01
312.5	3.60 ± 0.34	3.11 ± 0.45	0.66 ± 0.07	0.63 ± 0.10	0.21 ± 0.02	0.18 ± 0.02	8.61 ± 0.70	6.90 ± 0.93	0.61 ± 0.14	0.58 ± 0.15	0.33 ± 0.02	0.30 ± 0.04	0.64 ± 0.11	0.04 ± 0.01
NC	3.27 ± 0.41	3.05 ± 0.23	0.61 ± 0.03	0.58 ± 0.07	0.20 ± 0.01	0.18 ± 0.03	9.29 ± 1.27	6.77 ± 0.62	0.66 ± 0.16	0.58 ± 0.07	0.31 ± 0.02	0.31 ± 0.05	0.65 ± 0.14	0.05 ± 0.01

Organ coefficient = organ weight/body weight × 100%. The data were compared with the same sex of control group. *Significantly different from the NC group (*p* < 0.05). **♀-**female**, ♂-**male.

Taken together, these results indicate that dietary administration of SREC at doses up to 50,000 mg/kg feed for 90 days produced no observable subchronic toxicity in rats.

### 3. Salmonella reverse mutation (Ames) test

**Figure 4:**
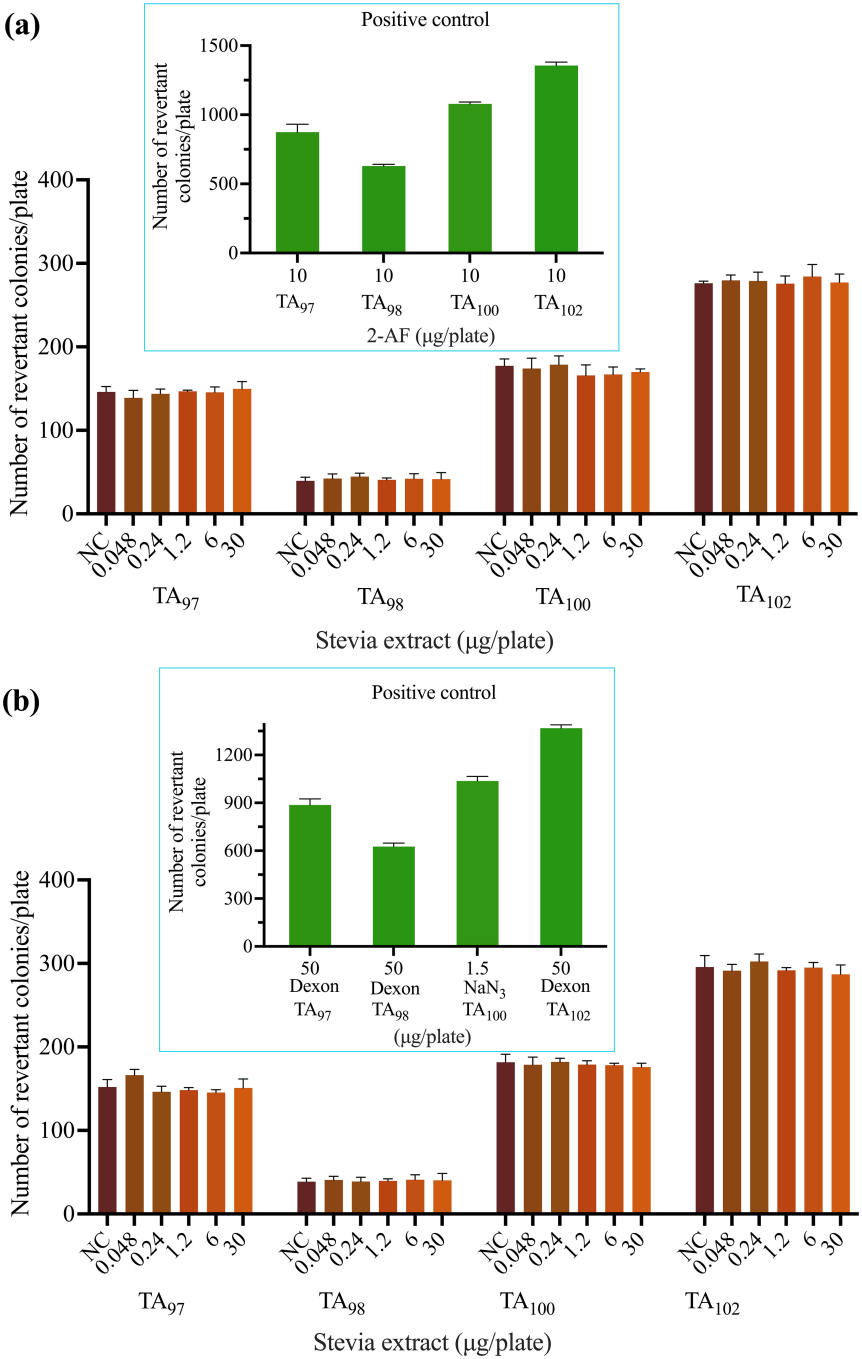
Effect of SREC on bacterial reverse mutation assay (Ames test). **(a)** With (+ S9 mix) and **(b)** without (− S9 mix) metabolic activation. 2-AF: 2-aminofluorene. Dexon: Fenaminosulf. NaN_3_: Sodium azide. NC: negative control.

The mutagenic potential of SREC was evaluated using the Ames test, a widely accepted method for assessing genetic toxicity. Results from two independent experiments using *Salmonella typhimurium* strains TA_97_, TA_98_, TA_100_, and TA_102_ are presented in Figure 4 and Supplementary Figure S3. As shown in Figures 4a,b, all strains exhibited normal bacterial lawn morphology. The positive controls were valid, producing a significant increase (*p* < 0.05) in revertant colonies—exceeding four times the number observed in the negative control.

SREC was tested at concentrations ranging from 0.048 to 30 μg/plate under both S9-activated and non-activated conditions. At all doses, the number of revertant colonies remained statistically comparable to that of the negative control group (*p* > 0.05), indicating no mutagenic effect.

Therefore, SREC was considered non-mutagenic under the conditions of the Ames test.

### Micronucleus test on bone marrow cells of mice

The micronucleus assay was conducted to evaluate potential chromosomal damage. Results of the bone marrow micronucleus test in female and male mice are presented in Table 4. As shown, the frequency of micronucleated erythrocytes in the positive control group was significantly higher than in the negative control group (*p* < 0.01). No significant differences in micronucleus frequency were observed between any SREC-treated group and the negative control in either sex (*p* > 0.05). The ratio of polychromatic erythrocytes (PCE) to mature erythrocytes (RBC) remained within the normal range across all groups. These findings indicate that SREC did not induce micronucleus formation in bone marrow cells at doses of 1,250–5,000 mg/kg·bw.

**Table 4:** The results of the micronucleus test on mouse bone marrow cells using SREC.

Groups (mg/kg·bw)	PCE/RBC ratio		MN/1000 PCE (%)		Significance of difference
	**♂**	**♀**	**♂**	**♀**	
5,000	0.99 ± 0.10	0.97 ± 0.08	5.17 ± 2.37	4.14 ± 2.39	*p* > 0.05
2,500	0.96 ± 0.05	1.00 ± 0.06	4.54 ± 1.92	5.18 ± 2.25	*p* > 0.05
1,250	1.01 ± 0.09	0.97 ± 0.06	4.78 ± 1.61	5.48 ± 2.01	*p* > 0.05
NC	0.97 ± 0.08	0.99 ± 0.05	5.15 ± 1.98	4.95 ± 1.85	-
PC	0.78 ± 0.08	0.87 ± 0.14	21.06 ± 4.63	19.89 ± 7.29	*p* < 0.01**

The data were compared with the negative control group, *Significantly different from the NC group (*p* < 0.05), **Significantly different from the NC group (*p* < 0.01). **♀-**female**, ♂-**male.

### Sperm abnormality test in mice

The sperm abnormality assay was conducted to assess potential germ cell genetic toxicity. Sperm abnormality rates for each experimental group are presented in Table 5, with the distribution of specific malformations shown in Supplementary Figure S4. As indicated in Table 5, the sperm abnormality rate in the positive control group was significantly higher than that in the negative control group (*p* < 0.01). In contrast, no significant increase in sperm abnormalities was observed in SREC-treated mice compared to the negative control (*p* > 0.05), indicating that SREC had no significant effect on sperm morphology at doses of 1,250–5,000 mg/kg·bw.

**Table 5:** Results of sperm malformation test in mice with SREC (sperm malformation rate).

Groups (mg/kg·bw)	Malformed sperm count	Malformed sperm count (%)	Significance of difference
5,000	152	3.03 ± 0.65	*p* > 0.05
2,500	139	2.78 ± 0.79	*p* > 0.05
1,250	145	2.90 ± 0.74	*p* > 0.05
NC	133	2.66 ± 0.68	--
PC	312	6.24 ± 0.90	*p* < 0.01**

The number of mice in each dose group was 5 and the sperm count was 5,000; the data were compared with the negative control group, *Significantly different from the NC group (*p* < 0.05), **Significantly different from the NC group (*p* < 0.01).

### Teratogenicity test in rats: reproductive and embryotoxicity

**Figure 5:**
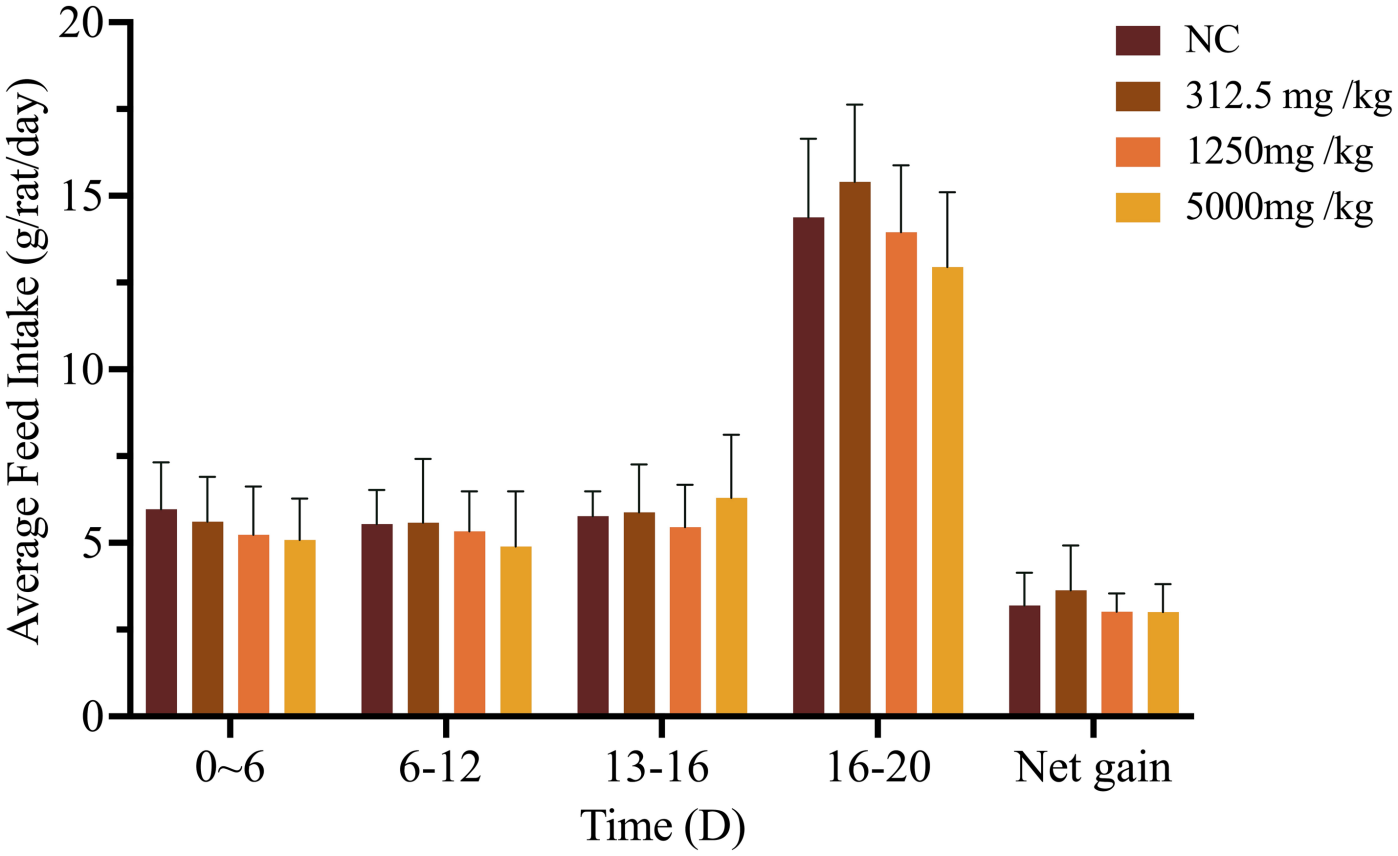
Effects of SREC on daily weight gain in pregnant rats. Net weight gain = gestational mouse weight gain – litter weight. *Significantly different from the NC at *p* < 0.05, **Significantly different from the NC at *p* < 0.01.

Throughout the study period, no clinical signs of toxicity or mortality were observed in pregnant rats administered SREC at doses of 312.5, 1,250, or 5,000 mg/kg·bw, nor in those in the negative control group. As shown in Figure 5, maternal weight gain did not differ significantly among treatment groups compared to controls (*p* > 0.05), indicating that SREC did not adversely affect maternal health within the tested dose range.

On gestation Day 20, dams were sacrificed, and reproductive parameters—including ovarian weight, number of corpora lutea, implantation sites, uterine weight, and number of live fetuses—were evaluated (Table 6). Fetal outcomes such as live birth rate, stillbirth rate, resorption rate, placental weight, fetal weight, body length, and tail length were also assessed (Tables 6, 7). Across all treatment groups, no statistically significant differences were found in reproductive or fetal developmental parameters compared to controls (*p* > 0.05).

**Table 6:** Effects of SREC on reproductive function in rats.

Groups (mg/kg·bw)	Ovarian weight(g)	Corpus luteum number	Average number of plants	Uterine weight (g)	Average number of live births
5,000	0.18 ± 0.02	16.08 ± 3.15	14.08 ± 2.47	6.91 ± 0.59	14.00 ± 2.30
1,250	0.18 ± 0.03	16.92 ± 1.68	13.67 ± 1.30	7.51 ± 0.53	13.58 ± 1.38
312.5	0.21 ± 0.02	17.33 ± 1.07	14.25 ± 1.54	7.05 ± 0.40	14.67 ± 1.83
NC	0.19 ± 0.05	17.08 ± 1.83	15.17 ± 1.64	7.56 ± 0.65	15.33 ± 1.92

The data in the table are the average of 12 pregnant rats in each group.

**Table 7:** Effects of SREC on embryonic development of rats.

Groups (mg/kg·bw)	Number of live births	Placental weight (g)	Fetal rat weight (g)	Fetal length (cm)	Fetal tail length (cm)
5,000	168	0.52 ± 0.02	3.68 ± 0.09	3.67 ± 0.06	1.21 ± 0.05
1,250	163	0.51 ± 0.02	3.65 ± 0.05	3.67 ± 0.02	1.21 ± 0.04
312.5	171	0.52 ± 0.01	3.64 ± 0.07	3.67 ± 0.03	1.22 ± 0.02
NC	182	0.51 ± 0.02	3.68 ± 0.06	3.68 ± 0.04	1.22 ± 0.05

The data in the table are the average of 12 pregnant rats in each group. *Significantly different from the NC group (*p* < 0.05), **Significantly different from the NC group (*p* < 0.01).

**Figure 6:**
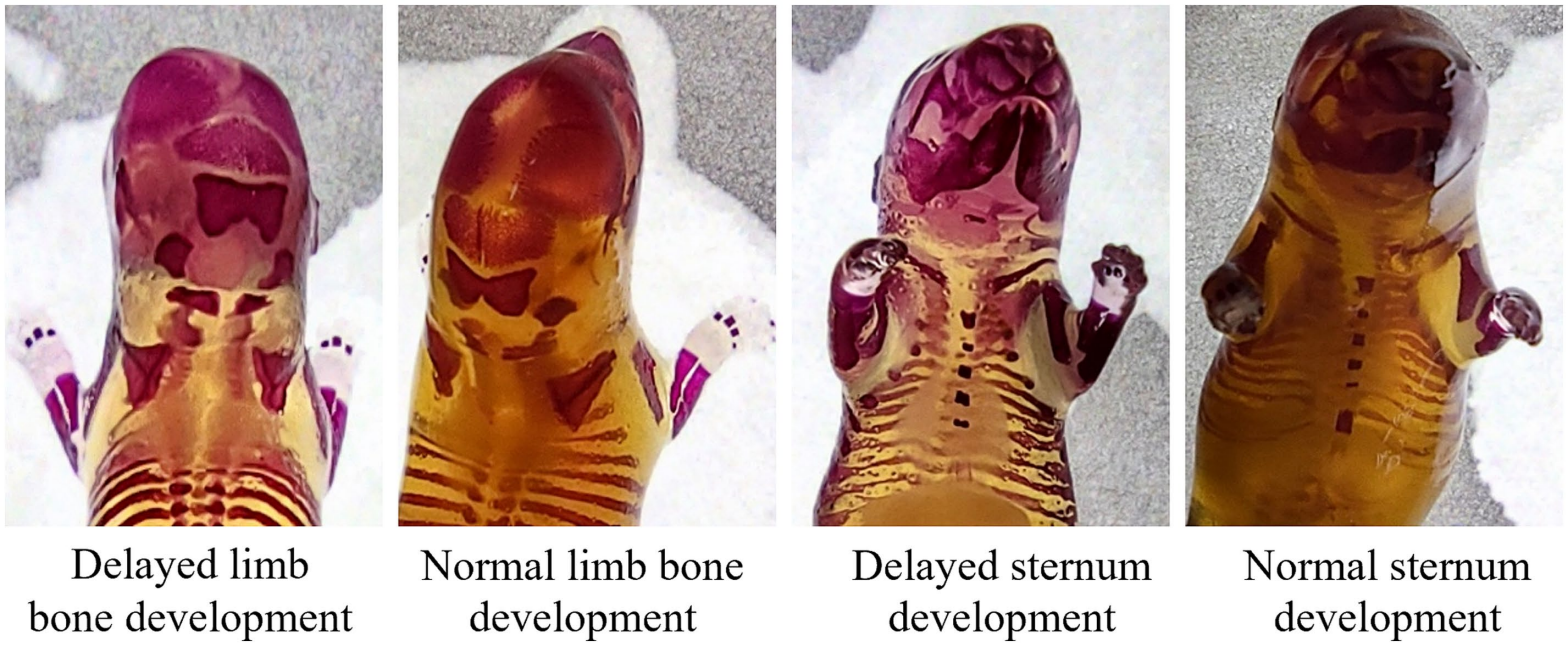
Effect of plant soot on skeletal malformations in rats.

Malformation assessments—including external, skeletal, and visceral examinations—revealed no adverse effects attributable to SREC. No external anomalies were observed in any group (Table S5). Skeletal analysis showed mild ossification delays in both treated and control fetuses, primarily involving the sternum and limb bones, with no significant differences in skeletal malformation rates (Supplementary Table S6 and Figure 6). No visceral abnormalities were detected (Supplementary Table S7).

Taken together, these findings demonstrate that SREC does not induce maternal toxicity, reproductive toxicity, embryotoxicity, or teratogenic effects in rats at doses up to 5,000 mg/kg·bw, as evidenced by normal reproductive performance, fetal development, and morphological integrity across all examined endpoints.

## Discussion

The long history of herbal medicine suggests that traditional herbs are generally regarded as non-toxic and clinically effective. However, the safety of these herbal substances remains insufficiently studied. SREC, renowned for its potent antioxidant and anti-inflammatory properties, has shown promising benefits in improving growth performance and boosting health across various animal species. Nevertheless, the potential systemic toxicity of SREC has not been comprehensively evaluated. Therefore, this study aimed to fill this gap by conducting a full toxicological evaluation of SREC, including assessments of acute oral toxicity, sub-chronic toxicity, genotoxicity, and teratogenicity.

In terms of acute toxicity, SREC was assessed by administering 5,000 mg/kg·bw to rats and mice over a 14-day observation period (30). No clinical symptoms or toxic deaths were observed, and no abnormal findings were recorded during necropsy. These findings confirm the absence of acute toxicity and align with findings from similar studies on other plant-based feed additives. For example, an acute toxicity study on *Campomanesia velutina* aqueous extract found no significant toxic effects at doses up to 1,200 mg/kg·bw in mice, with only transient symptoms, such as diarrhea, abdominal cramps, and tremors, observed at the highest dose (17). Similarly, the hydroalcoholic extract of *Withania somnifera* roots, when administered at doses up to 2,000 mg/kg·bw in an acute toxicity test, did not cause any toxic symptoms, mortality, or behavioral changes in rats during the 14-day observation period (16). The findings from this study reinforce the conclusion that SREC exhibits no acute toxic potential at high dose.

Following the acute toxicity evaluation, a 90-day subchronic toxicity study was conducted using dietary SREC at doses ranging from 2,000 to 50,000 mg/kg feed. No significant changes in behavior, feed intake, or body weight were observed, and there were no abnormalities detected in organ system toxicity evaluations, including the heart, liver, kidneys, and gastrointestinal tract. Hematological and biochemical parameters remained within normal physiological ranges, with no dose-dependent effects.

These observations are consistent with previous findings on other plant-based feed additives. For example, an investigation of *Arecae semen* aqueous extract showed that at doses up to 4,500 mg/kg feed, no significant changes were observed in biochemical parameters or organ weights in rats, although some mild signs of toxicity, including weight loss and changes in liver and testis organ weights, were observed in the high-dose group (31). Similarly, a study on marigold flavonoids from marigold inflorescence residue, administered at doses up to 50,000 mg/kg feed for 90 days, showed no significant adverse effects on body weight or organ function (18). In contrast, the sub-chronic toxicity study on *Nityanand Rasa* (NR) (19), an Ayurvedic herbo-metallic formulation, showed mild liver and kidney toxicity at high doses of 600 mg/kg feed. These comparisons collectively suggest that SREC may have a broader safety margin than some other plant-based formulations, especially at higher doses.

To further assess genetic safety, SREC was evaluated using the Ames test, micronucleus assay, and sperm morphology test, and it did not induce any statistically significant abnormalities in any of these assays (32–34). This consistent lack of genotoxic responses across multiple endpoints provides robust evidence for the genetic safety of SREC (35). These findings are consistent with other studies of herbal extracts. For instance, a study on marigold flavonoids, extracted from marigold inflorescence residue, showed no evidence of genotoxicity in the Ames test, sperm aberration test, or *in vivo* micronucleus test at doses up to 5,000 mg/kg·bw (18). Similarly, a study on *Agrimonia* and *Filipendula* species plant extracts demonstrated no mutagenic or genotoxic effects in both the Ames test and micronucleus assay, supporting the non-genotoxic potential of these herbal extracts (23). By contrast, Metolcarb, an insecticide, produced strong genotoxicity responses, including increased mutagenic revertants and chromosomal damage in various test systems. These comparisons further emphasize the favorable genetic safety profile of SREC.

In addition to systemic and genetic toxicity, potential reproductive and developmental toxicity of SREC was assessed via a teratogenicity study (36). This evaluation was designed to detect potential effects on fertility, fetal development, and neonatal outcomes (37). In the teratogenicity study, no significant effects were observed on reproductive function, fetal development, or birth outcomes at doses ranging from 312.5 to 5,000 mg/kg·bw. There were no significant differences in reproductive indicators such as ovarian weight, implantation number, and live birth rates between the treated groups and the negative control group. Similarly, fetal growth and development indicators, including placental weight, fetal weight, body length, and tail length, remained unaffected. These outcomes strongly support the absence of teratogenic effects, indicating that SREC is unlikely to impair reproductive performance or fetal health. Therefore, its application in breeding animal diets appears to be safe and without detrimental effects on progeny development.

Despite these positive results, several limitations should be acknowledged. The 90-day duration and limited sample size may not fully capture the potential long-term effects of SREC. Future studies should consider extended exposure periods and larger populations to validate these findings. Additionally, the current genotoxicity and teratogenicity assessments only represent specific toxicological endpoints. Additional evaluations involving neurotoxicity, endocrine-disrupting potential, and immunotoxicity would be beneficial to comprehensively characterize the safety profile of SREC.

In summary, this study provides foundational evidence supporting the safety of SREC, demonstrating no acute, sub-chronic, genotoxic, or teratogenic toxicity. These findings support its potential use as a safe and effective natural feed additive. However, further research is necessary to elucidate the mechanisms of action of its bioactive components, particularly chlorogenic acid and its derivatives—especially in relation to immune modulation, cellular signaling, and gut microbiota composition. Moreover, long-term safety assessments across multiple species and production contexts are essential. Given its favorable characteristics, SREC deserves further investigation as a potential substitute for antibiotic growth promoters in livestock farming (38, 39).

## Conclusion

This study provides a comprehensive assessment of the safety profile of SREC, utilizing various *in vivo* and *in vitro* tests. The acute oral toxicity test demonstrated that SREC is well-tolerated, with no adverse effects or mortality at doses up to 5,000 mg/kg·bw (LD_50_ > 5,000 mg/kg·bw). The 90-day repeated dose oral toxicity study confirmed the safety of SREC at doses up to 50,000 mg/kg feed, with no significant changes in vital signs, behavior, hematological or biochemical indices, immune function, or organ health. Additionally, genotoxicity assessments, including the Ames test, mouse bone marrow micronucleus assay, and sperm morphology test, showed that SREC does not exhibit genotoxic properties. Teratogenicity testing on pregnant rats revealed no significant teratogenic effects, with no observed differences in embryonic development compared to the negative control group.

These findings collectively support the safety of SREC as a promising natural feed additive for livestock and poultry. However, while the results are encouraging, several limitations in this study must be acknowledged. The sample size and the duration of exposure were limited, and while no adverse effects were observed in the experimental animals, further studies with larger sample sizes and longer durations are needed to more comprehensively evaluate the long-term safety and efficacy of SREC. Additionally, future research should include testing in other animal models and under varying environmental conditions to confirm the generalizability of these results.

In conclusion, SREC demonstrates a favorable safety profile and holds significant potential as a sustainable alternative to traditional antibiotics in animal feed. Further research is necessary to fully establish its long-term safety and efficacy across a broader range of applications.

## Data Availability

The dataset generated and analyzed during the current study is available from the corresponding author on reasonable request. There are Requests to access the datasets should be directed to lytingm@163.com.
