# Supplementary material for: Stevia rebaudiana extract (main components: chlorogenic acid and its analogues) as a new safe feed additive: evaluation of acute toxicity, sub chronic toxicity, genotoxicity, and teratogenicity
Source: Front Vet Sci. 2025 Sep 4;12:1646665. doi: 10.3389/fvets.2025.1646665 (PMC12444892; doi:10.3389/fvets.2025.1646665)
Supplement: Supplementary file 1 [file Image_1.pdf]

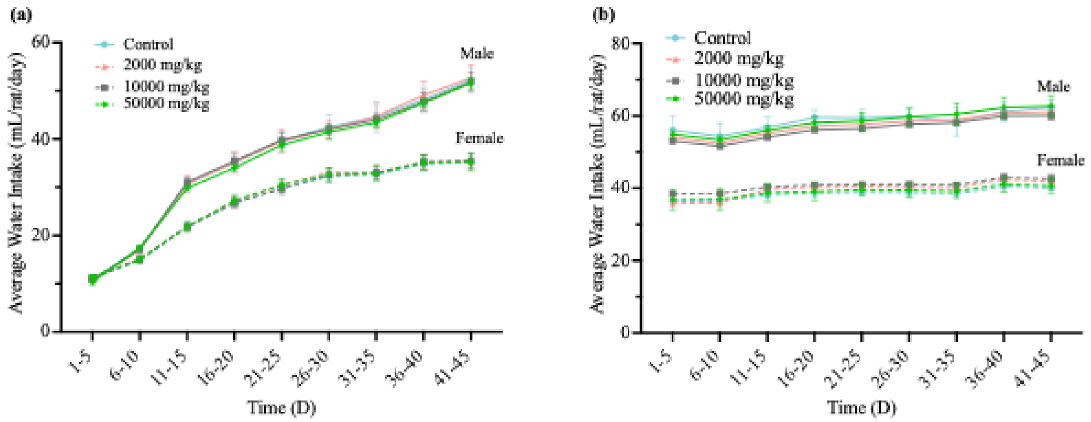

Fig. 1 Effect of stevia extract on average water drink of rats in the 90-day feeding study, as follows: (a) 1–45 days water drink, (b) 46–90 days water drink, no significant difference was observed. The dosage units are all: mg/kg feed.
