# Supplementary material for: Stevia rebaudiana extract (main components: chlorogenic acid and its analogues) as a new safe feed additive: evaluation of acute toxicity, sub chronic toxicity, genotoxicity, and teratogenicity
Source: Front Vet Sci. 2025 Sep 4;12:1646665. doi: 10.3389/fvets.2025.1646665 (PMC12444892; doi:10.3389/fvets.2025.1646665)
Supplement: Supplementary file 2 [file Image_2.pdf]

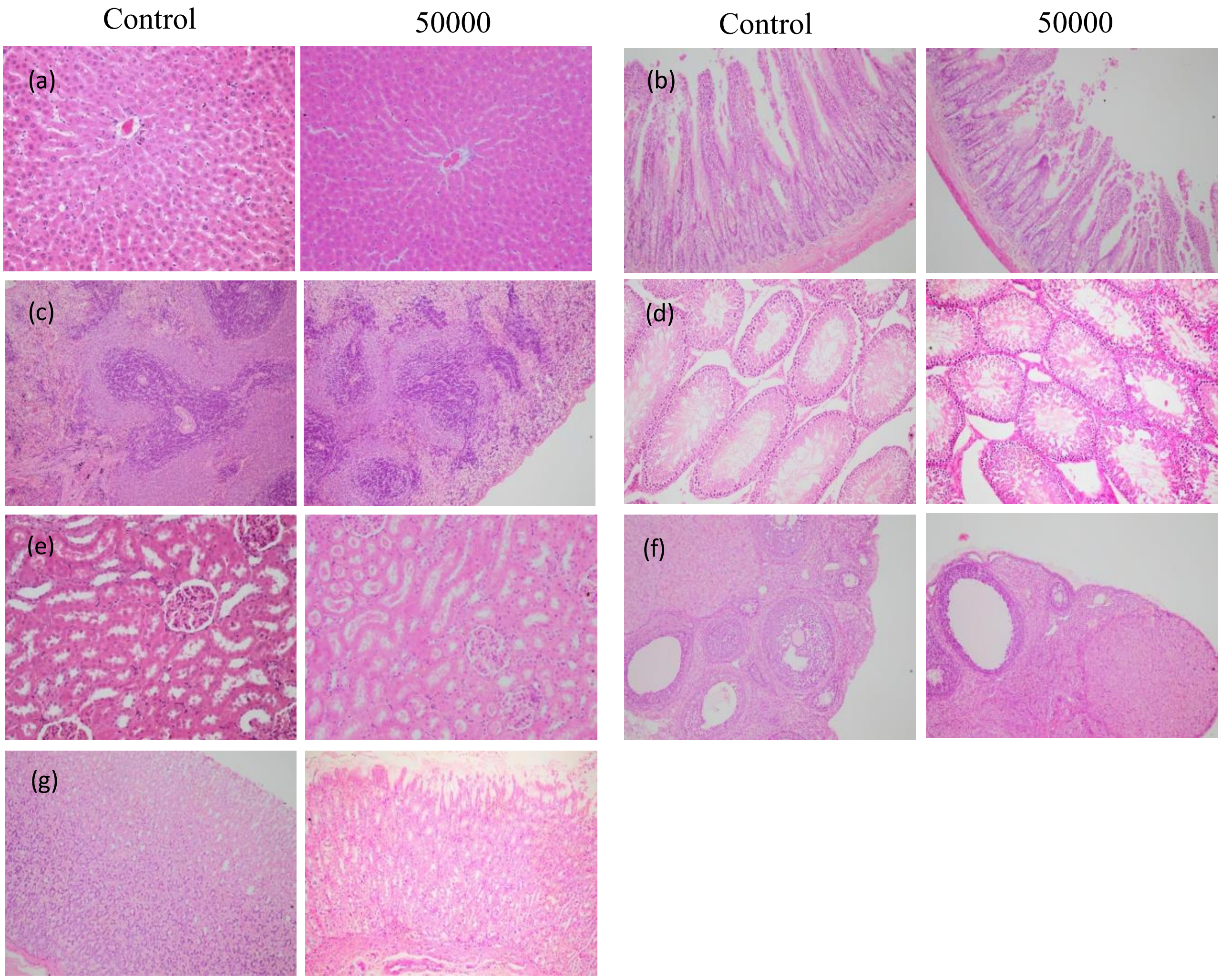

Fig. 2 Effect of Stevia extract on the histopathological change in rats after feeding for 45 days (female and male were similar, only the control group and the high-dose group were included); (A) liver; (B) intestine; (C) spleen; (D) testis; (E) kidney; (F) ovary; (G) stomach.
