# Supplementary material for: Stevia rebaudiana extract (main components: chlorogenic acid and its analogues) as a new safe feed additive: evaluation of acute toxicity, sub chronic toxicity, genotoxicity, and teratogenicity
Source: Front Vet Sci. 2025 Sep 4;12:1646665. doi: 10.3389/fvets.2025.1646665 (PMC12444892; doi:10.3389/fvets.2025.1646665)
Supplement: Supplementary file 3 [file Image_3.pdf]

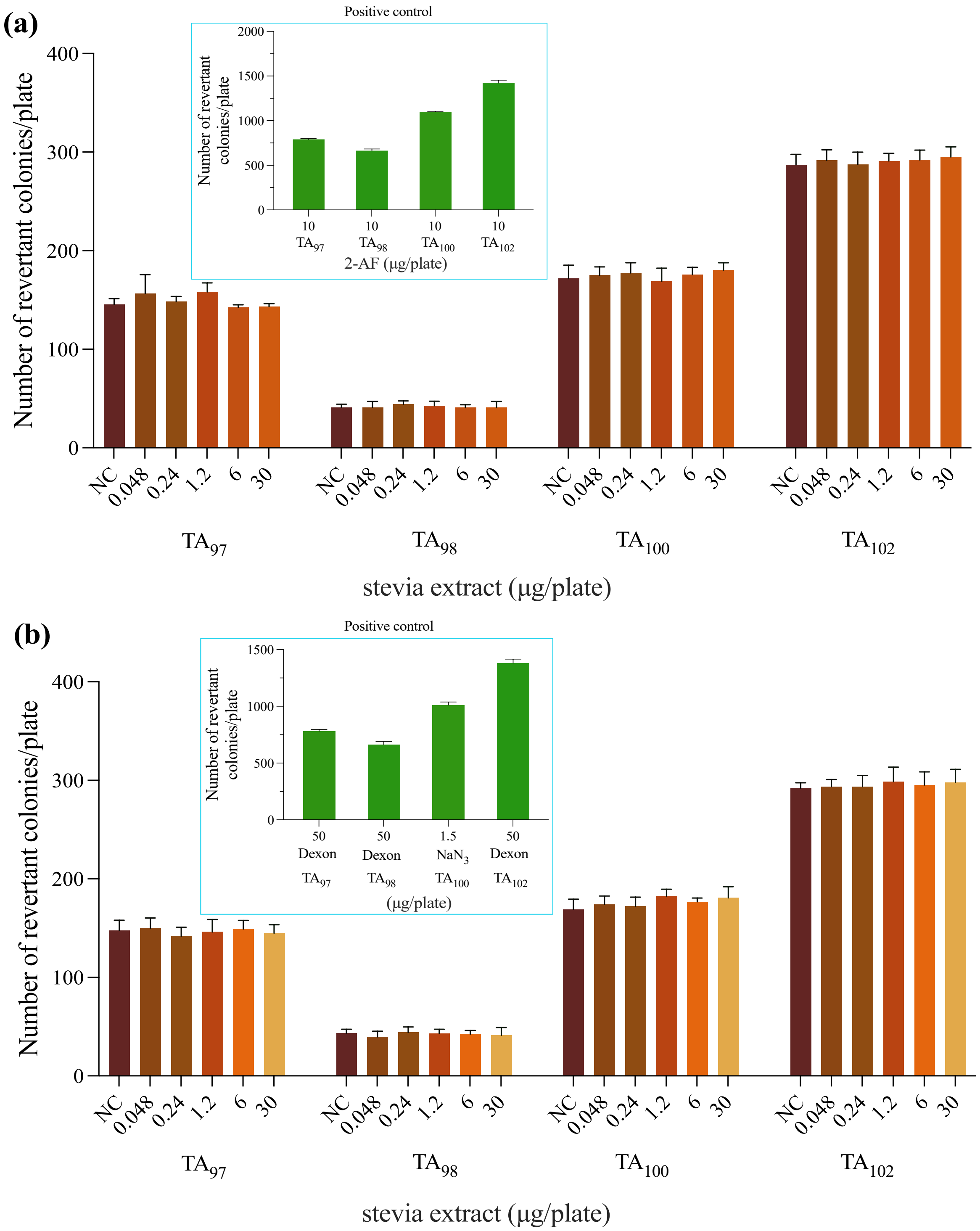

Fig.3 Effect of stevia extract on bacterial reverse mutation assay (Ames test). (a) with (+S9mix) and (b) without (-S9mix) metabolic activation. 2-AF:2-amino fluorene. Dexon: Fenaminosulf. NaN_3: Sodium azide. NC: negative control.
