# Supplementary material for: Stevia rebaudiana extract (main components: chlorogenic acid and its analogues) as a new safe feed additive: evaluation of acute toxicity, sub chronic toxicity, genotoxicity, and teratogenicity
Source: Front Vet Sci. 2025 Sep 4;12:1646665. doi: 10.3389/fvets.2025.1646665 (PMC12444892; doi:10.3389/fvets.2025.1646665)
Supplement: Supplementary file 5 [file Table_1.docx]

**Table 1** Feeding Stevia Extract for 45 Days: Effects on Hematological Parameters in SD Rats

| **Groups**  **(mg/kg**  **feed)** | **HGB（g/L）** | | **RBC (10^12^/L)** | | **WBC (10^9^/L)** | | **PLT (10^9^/L)** | | **HCT (%)** | | **EOS (10^9^/L)** | | **BAS (10^9^/L)** | | **NEU (10^9^/L)** | | **MO (10^9^/L)** | | **LYM (10^9^/L)** | |
| --- | --- | --- | --- | --- | --- | --- | --- | --- | --- | --- | --- | --- | --- | --- | --- | --- | --- | --- | --- | --- |
|  | ♀ | ♂ | ♀ | ♂ | ♀ | ♂ | ♀ | ♂ | ♀ | ♂ | ♀ | ♂ | ♀ | ♂ | ♀ | ♂ | ♀ | ♂ | ♀ | ♂ |
| 50000 | 150.40±8.17 | 157.80±8.32 | 6.81±0.41 | 7.17±0.18 | 9.07±0.74^＊^ | 9.18±1.57 | 739.60±93.23 | 771.00±79.57 | 45.26±2.64 | 47.10±2.69 | 0.04±0.03 | 0.02±0.01 | 0±0 | 0±0 | 2.76±0.52 | 2.90±0.83 | 1.08±0.28^＊^ | 1.13±0.31 | 5.19±0.38 | 5.13±0.76 |
| 10000 | 171.20±13.27^＊^ | 153.40±4.39^＊^ | 7.52±0.37^＊^ | 7.23±0.13 | 9.69±2.16^＊^ | 9.33±1.16 | 741.80±73.25 | 773.60±112.28 | 48.40±2.13^＊^ | 44.60±1.03^＊^ | 0.03±0.01 | 0.05±0.06 | 0±0 | 0±0 | 3.49±0.52^＊^ | 3.62±1.18 | 1.00±0.36^＊^ | 0.58±0.12^＊^ | 5.16±1.92 | 5.08±0.72 |
| 2000 | 155.60±10.01 | 156.80±23.20 | 7.12±0.37 | 7.17±0.92 | 8.82±3.31 | 7.78±1.03 | 725.00±33.00 | 645.61±46.97 | 45.10±2.94 | 46.26±6.87 | 0.03±0.01 | 0.03±0.01 | 0±0 | 0±0 | 2.49±0.79 | 1.86±0.35^＊^ | 0.88±0.38 | 0.84±0.22 | 5.42±2.57 | 5.05±0.93 |
| NC | 153.20±6.83 | 165.00±6.78 | 7.00±0.28 | 7.59±0.40 | 6.48±2.08 | 8.77±1.06 | 770.40±112.80 | 776.80±95.07 | 45.22±1.56 | 48.12±1.81 | 0.03±0.02 | 0.03±0.02 | 0±0 | 0±0 | 2.02±0.99 | 3.25±0.83 | 0.48±0.21 | 0.84±0.18 | 3.95±1.30 | 4.65±1.32 |

**Note:** **﻿***Significantly different from the NC at *P* < 0.05, **﻿****Significantly different from the NC at *P* < 0.01. ♀: female, ♂: male.
