# Supplementary material for: Stevia rebaudiana extract (main components: chlorogenic acid and its analogues) as a new safe feed additive: evaluation of acute toxicity, sub chronic toxicity, genotoxicity, and teratogenicity
Source: Front Vet Sci. 2025 Sep 4;12:1646665. doi: 10.3389/fvets.2025.1646665 (PMC12444892; doi:10.3389/fvets.2025.1646665)
Supplement: Supplementary file 6 [file Table_2.docx]

**Table 2** Effects of Stevia Extract on Blood Biochemical Parameters in SD Rats After 45 Days of Feeding

| **Groups**  **(mg/kg**  **feed)** | **Alb(g/L)** | | | **ALT(U/L)** | | **AST(U/L)** | | | **TCH (mmol/L)** | | **Cr (μmol/L)** | | **Glu(mmol/L)** | | | **TG (mmol/L)** | | | **TP(g/L)** | | **BUN (mmol/L)** | |
| --- | --- | --- | --- | --- | --- | --- | --- | --- | --- | --- | --- | --- | --- | --- | --- | --- | --- | --- | --- | --- | --- | --- |
|  | ♀ | ♂ | | ♀ | ♂ | ♀ | ♂ | | ♀ | ♂ | ♀ | ♂ | ♀ | | ♂ | ♀ | ♂ | | ♀ | ♂ | ♀ | ♂ |
| 50000 | 39.62±1.29^＊^ | | 38.4±0.63 | 47.20±0.84^＊^ | 51.20±3.77^＊^ | 98.40±12.62 | 98.20±6.53 | 1.65±0.29 | | 2.14±0.39 | 41.97±9.58 | 44.23±13.56 | 10.04±0.69^＊^ | 9.58±1.87 | | 1.54±0.49^＊^ | | 1.20±0.34 | 63.16±1.58^＊^ | 60.34±2.05 | 5.94±0.34 | 5.11±0.56 |
| 10000 | 40.82±0.94 | | 39.3±1.59 | 53.20±7.26 | 50.20±4.49^＊^ | 114.40±12.88 | 95.80±7.98 | 1.99±0.10 | | 2.36±0.53 | 38.87±5.62 | 32.59±4.61 | 10.02±1.00^＊^ | 10.72±2.80 | | 1.14±0.38 | | 1.12±0.41 | 64.36±2.81 | 63.78±3.30 | 6.16±0.80 | 5.88±1.02 |
| 2000 | 41.58±2.42 | | 40.1±1.08 | 52.20±8.76 | 49.60±7.64^＊^ | 106.40±5.77 | 111.40±14.10 | 2.36±0.17 | | 2.13±0.26 | 41.33±7.28 | 36.60±7.72 | 9.06±1.09^＊^ | 8.30±1.75 | | 0.81±0.28 | | 0.83±0.25 | 66.40±3.06 | 65.48±1.26 | 5.78±0.26 | 5.20±0.43 |
| NC | 41.72±0.91 | | 40.1±1.90 | 64.40±7.80 | 60.40±4.51 | 107.40±13.69 | 106.60±7.37 | 1.98±0.40 | | 2.06±0.14 | 44.28±10.16 | 37.04±4.21 | 7.68±0.45 | 9.68±1.80 | | 0.73±0.14 | | 0.83±0.25 | 67.08±1.67 | 63.50±3.47 | 6.35±0.64 | 5.69±0.46 |

**Note:** **﻿***Significantly different from the NC at *P* < 0.05, **﻿****Significantly different from the NC at *P* < 0.01. ♀: female, ♂: male.
