# Supplementary material for: Stevia rebaudiana extract (main components: chlorogenic acid and its analogues) as a new safe feed additive: evaluation of acute toxicity, sub chronic toxicity, genotoxicity, and teratogenicity
Source: Front Vet Sci. 2025 Sep 4;12:1646665. doi: 10.3389/fvets.2025.1646665 (PMC12444892; doi:10.3389/fvets.2025.1646665)
Supplement: Supplementary file 7 [file Table_3.docx]

**Table 3** Effects of Stevia Extract on Organ Coefficients in SD Rats After 45 Days of Feeding

| **Groups**  **(mg/kg feed)** | **Liver** | | **Kidney** | | **Spleen** | | **Gastrointestinal** | | **Lung** | | **Heart** | | **Testicle** | **Ovary** |
| --- | --- | --- | --- | --- | --- | --- | --- | --- | --- | --- | --- | --- | --- | --- |
|  | ♀ | ♂ | ♀ | ♂ | ♀ | ♂ | ♀ | ♂ | ♀ | ♂ | ♀ | ♂ |  |  |
| 5000 | 3.21±0.40 | 3.19±0.25 | 0.70±0.19 | 0.70±0.05 | 0.27±0.04 | 0.19±0.03 | 8.70±1.73 | 8.16±1.19 | 0.79±0.14 | 0.87±0.26 | 0.37±0.04 | 0.33±0.03 | 0.80±0.05 | 0.05±0.01 |
| 1250 | 3.39±0.18 | 3.45±0.18 | 0.71±0.06 | 0.70±0.03 | 0.25±0.02 | 0.22±0.02 | 8.73±0.99 | 7.25±0.92 | 1.02±0.25 | 0.81±0.21 | 0.38±0.02 | 0.34±0.03 | 0.77±0.09 | 0.06±0.01 |
| 312.5 | 3.19±0.45 | 3.11±0.45 | 0.78±0.10 | 0.76±0.09 | 0.25±0.04 | 0.20±0.03 | 7.74±0.62 | 6.90±0.93 | 1.01±0.28 | 0.77±0.04 | 0.33±0.03 | 0.36±0.06 | 0.82±0.12 | 0.06±0.01 |
| NC | 3.29±0.09 | 3.25±0.23 | 0.67±0.07 | 0.69±0.06 | 0.23±0.05 | 0.21±0.03 | 7.59±0.74 | 7.05±0.55 | 0.75±0.15 | 0.80±0.08 | 0.36±0.05 | 0.32±0.03 | 0.79±0.08 | 0.06±0.01 |

**Note:** **﻿***Significantly different from the NC at *P* < 0.05, **﻿****Significantly different from the NC at *P* < 0.01. ♀: female, ♂: male.
