# Supplementary material for: Stevia rebaudiana extract (main components: chlorogenic acid and its analogues) as a new safe feed additive: evaluation of acute toxicity, sub chronic toxicity, genotoxicity, and teratogenicity
Source: Front Vet Sci. 2025 Sep 4;12:1646665. doi: 10.3389/fvets.2025.1646665 (PMC12444892; doi:10.3389/fvets.2025.1646665)
Supplement: Supplementary file 8 [file Table_4.docx]

**Table 4** Effect of stevia extract on the survival rate of rat embryo

| **Groups(mg/kg) bw** | **Number of pregnant rats** | **Implantation rate (%)** | **Number of live fetuses (♀/♂)** | **Resorption rate (%)** | **Dead fetus rate (%)** | **Live fetus rate (%)** |
| --- | --- | --- | --- | --- | --- | --- |
| 5000 | 12 | 88.02 | 86/82 | 0.59(1/168) | 0(0/168) | 99.41(167/168) |
| 1250 | 12 | 83.25 | 76/87 | 0.61(1/163) | 0(0/163） | 99.39(162/163) |
| 312.5 | 12 | 86.27 | 90/81 | 2.84(5/171) | 0(0/177) | 97.16(166/171) |
| NC | 12 | 92.46 | 88/94 | 1.09(2/182) | 0 (0/182) | 98.91(180/182) |

**Note:** The data in the table are the average of 12 pregnant mice in each group (means ± SD). Comparisons were made with the negative control group. Note: **﻿***Significantly different from the NC at *P* < 0.05, **﻿****Significantly different from the NC at *P* < 0.01. Absorption rate (%) = number of absorbed fetuses / number of implantations; stillbirth rate (%) = amount of stillbirths / number of implantations; live birth rate (%) = number of live fetuses / number of implantations. ♀: female, ♂: male.
