# Supplementary material for: Stevia rebaudiana extract (main components: chlorogenic acid and its analogues) as a new safe feed additive: evaluation of acute toxicity, sub chronic toxicity, genotoxicity, and teratogenicity
Source: Front Vet Sci. 2025 Sep 4;12:1646665. doi: 10.3389/fvets.2025.1646665 (PMC12444892; doi:10.3389/fvets.2025.1646665)
Supplement: Supplementary file 9 [file Table_5.docx]

**Table 5** Appearance abnormality distribution of fetal rats in each experimental group

| **Groups(mg/kg) bw** | **number of fetal rats** | **Skin bleeding** | **Head abn.** | **Facial abn.** | **Trunk abn.** | **Forelimb abn.** | **Hindlimb abn.** | **Umbilical cord abn.** | **Tail abn.** | **Anus abn.** | **Urethral abn.** | **Total number of malformations** | **Average number of malformations** |
| --- | --- | --- | --- | --- | --- | --- | --- | --- | --- | --- | --- | --- | --- |
| 5000 | 168 | 0 | 0 | 0 | 0 | 0 | 0 | 0 | 0 | 0 | 0 | 0 | 0 |
| 1250 | 163 | 0 | 0 | 0 | 0 | 0 | 0 | 0 | 0 | 0 | 0 | 0 | 0 |
| 312.5 | 171 | 0 | 0 | 0 | 0 | 0 | 0 | 0 | 0 | 0 | 0 | 0 | 0 |
| NC | 182 | 0 | 0 | 0 | 0 | 0 | 0 | 0 | 0 | 0 | 0 | 0 | 0 |

**Note:** mean number of external malformations = total number of external malformations / number of fetuses examined. Abn.: abnormality.
