# Supplementary material for: Stevia rebaudiana extract (main components: chlorogenic acid and its analogues) as a new safe feed additive: evaluation of acute toxicity, sub chronic toxicity, genotoxicity, and teratogenicity
Source: Front Vet Sci. 2025 Sep 4;12:1646665. doi: 10.3389/fvets.2025.1646665 (PMC12444892; doi:10.3389/fvets.2025.1646665)
Supplement: Supplementary file 10 [file Table_6.docx]

**Table 6** Distribution of skeletal malformations in fetal rats in each experimental group

| **Groups(mg/kg)** | **number of fetal rats** | **Occipital abnormality** | **Abnormal vertebrae** | **Sternal anomaly** | **Rib anomaly** | **Pelvic anomaly** | **Abnormal bone of all four limbs** | **Total skeletal malformations** | **Mean skeletal deformity** |
| --- | --- | --- | --- | --- | --- | --- | --- | --- | --- |
| 5000 | 87 | 0 | 0 | 9 | 0 | 0 | 12 | 21 | 0.24 |
| 1250 | 85 | 0 | 0 | 7 | 0 | 0 | 8 | 15 | 0.17 |
| 312.5 | 89 | 0 | 0 | 10 | 0 | 0 | 14 | 24 | 0.27 |
| NC | 94 | 0 | 0 | 12 | 0 | 0 | 15 | 27 | 0.29 |

**Note:** mean number of skeletal malformations = total number of skeletal malformations / number of fetuses examined.
