# Supplementary material for: Stevia rebaudiana extract (main components: chlorogenic acid and its analogues) as a new safe feed additive: evaluation of acute toxicity, sub chronic toxicity, genotoxicity, and teratogenicity
Source: Front Vet Sci. 2025 Sep 4;12:1646665. doi: 10.3389/fvets.2025.1646665 (PMC12444892; doi:10.3389/fvets.2025.1646665)
Supplement: Supplementary file 11 [file Table_7.docx]

**Table 7** The distribution of fetal viscera malformation in each experimental group

| **Groups**  **(mg/kg bw)** | **number of live births** | **Brain abn.** | **Eye abn.** | **Tracheoesophageal abn.** | **Cardiac abn.** | **Pulmonary abn.** | **Abnormal liver** | **Nephrovesical anomaly** | **Genital organ abn.** | **Total Visceral malformations** | **Mean Visceral malformations** |
| --- | --- | --- | --- | --- | --- | --- | --- | --- | --- | --- | --- |
| 5000 | 81 | 0 | 0 | 0 | 0 | 0 | 0 | 0 | 0 | 0 | 0 |
| 1250 | 78 | 0 | 0 | 0 | 0 | 0 | 0 | 0 | 0 | 0 | 0 |
| 312.5 | 82 | 0 | 0 | 0 | 0 | 0 | 0 | 0 | 0 | 0 | 0 |
| NC | 88 | 0 | 0 | 0 | 0 | 0 | 0 | 0 | 0 | 0 | 0 |

**Note:** Mean number of visceral malformations = total number of visceral malformations/numbers of fetal rats examined. Abn: abnormality
